# Economic Analysis of a Pine Plantation Receiving Repeated Applications of Biosolids

**DOI:** 10.1371/journal.pone.0057705

**Published:** 2013-02-25

**Authors:** Hailong Wang, Mark O. Kimberley, Peter J. Wilks

**Affiliations:** 1 Zhejiang Provincial Key Laboratory of Carbon Cycling in Forest Ecosystems and Carbon Sequestration, and School of Environmental and Resource Sciences, Zhejiang Agricultural and Forestry University, Lin’an, Zhejiang Province, China; 2 Scion, Rotorua, New Zealand; 3 PF Olsen Ltd., Nelson, New Zealand; Tel Aviv University, Israel

## Abstract

Treated biosolids have been applied to 750-ha of a *Pinus radiata* forest plantation on Rabbit Island near Nelson City in New Zealand since 1996. A long-term research trial was established in 1997 to investigate the effects of the biosolids applications on the receiving environment and tree growth. An analysis of the likely economic impact of biosolids application shows that biosolids application has been beneficial. Stem volume of the high treatment (biosolids applied at 600 kg N ha^-1^ every three years) was 36% greater than the control treatment (no biosolids applied), and stem volume of the standard treatment (300 kg N ha^-1^) was 27% greater than the control treatment at age 18 years of age. Biosolids treatments have effectively transformed a low productivity forest site to a medium productivity site. Although this increased productivity has been accompanied by some negative influences on wood quality attributes with reduced wood stiffness, wood density, and larger branches, an economic analysis shows that the increased stem volume and greater average log diameter in the biosolids treatments outweighs these negative effects. The high and standard biosolids treatments are predicted to increase the net stumpage value of logs by 24% and 14% respectively at harvesting, providing a large positive impact on the forest owner’s economic return.

## Introduction

Biosolids are treated and stabilised sewage sludge that is suitable for beneficial use through land application. One of the common options for the management of biosolids in many parts of the world is its use as a fertiliser and soil amendment for improvement of low fertility soils and reclamation of degraded land [1], [2], [3]. Application of biosolids to land can enhance soil sequestration of carbon [4], [5], [6] as well as providing nutrients to crops [7]. Forest land application, rather than applying biosolids to agricultural land, can reduce the risk of contaminants entering the human food chain [8]. In addition, forest application can increase tree growth and subsequent economic returns [9], [10], [11], [12]. However, few studies have been conducted to quantify the economic return of forests associated with land application of biosolids.

Treated biosolids from the Nelson Regional Sewage Treatment Plant have been applied to 750-ha of a *Pinus radiata* D. Don plantation at Rabbit Island near Nelson City, New Zealand, since 1996. Repeated applications to individual forest stands within the plantation have been made approximately every three years. To investigate sustainability of the biosolids application programme, a long-term research trial was established in 1997. Tree growth responses have been measured along with a number of environmental variables, such as soil and groundwater quality [10], [13], [14], [15], [16].

Economic analysis based on the growth data at age 11 years (i.e., 5 years after trial establishment), indicated that trees in the standard biosolids rate treatment (300 kg N ha^-1^) had effectively gained 0.74 years-worth of volume growth over the controls, while those receiving the high rate biosolids treatment (600 kg N ha^-1^) had gained 1.10 years [17]. However, branch diameter of biosolids-treated trees had increased [17], and a small but significant reduction of basic wood density and standing-tree sonic velocity in *P. radiata* was also found in the biosolids treatments [10], which might reduce the potential value of the increased volume growth. Therefore, the objective of this study was to update predictions of the economic effects of applying biosolids to the plantation using growth measurements made at age 18 years, and measurements of log stiffness assessed using resonance velocity techniques at age 15 years.

## Materials and Methods

### Trial description

The trial was established in October 1997 in a stand of *P. radiata* which had been planted in 1991. Note that no specific permits were required for the described field studies and no specific permissions were required for the experimental activities on this plantation forest site that is for timber production. The location is not privately-owned or protected in any way. The field study did not involve endangered or protected species.

The soil at the trial site consists of coarse coastal dune sands deficient in nitrogen [13], classified as a sandy raw soil [18], which provides free rooting access to the shallow groundwater 2.0 to 4.2 m below the surface. The stand had been established at a stocking of 1000 stems ha^-1^, and all trees in the trial were pruned in up to four lifts to 6 m height during the period November 1996 to August 2001.

Three biosolids treatments were applied in a split-plot, randomised block design with four replicates. The treatments consisted of: a control (no biosolids); a standard treatment (target application of 300 kg N ha^-1^, equivalent to 100 kg N ha^-1^ year^-1^ over 3 years, as used operationally; and a high treatment (double the standard at a target rate of 600 kg N ha^-1^). Each main-plot contained three stocking density treatments (subplots) of approximately 300, 450, and 600 stems ha^-1^. There were 36 subplots (4 replicates×3 biosolids rates×3 stocking densities). Each subplot measured 25 m×25 m, plus 5 m buffer zones, so that the whole site covered an area of approximately 4 ha. Treated biosolids from the Nelson regional wastewater treatment plant were applied in 1997, 2000, 2003 and 2006.

Height and diameter at breast height (DBH) of all trees in each plot were measured annually from ages 7 to 18 years (from 1997 to 2009). All data were entered into the Forest Research Permanent Sample Plot System [19]. Stocking, mean top height (MTH, mean height of the largest 100 trees per hectare), basal area (BA), and stem volume were estimated for each subplot. Detailed descriptions of the methods for annual tree growth measurement, and statistical data analysis were described previously [10].

In June 2006 at age 15 years, one tree in each subplot was selected for destructive sampling generally from the plot buffer areas. Each tree was felled and then cross cut into 5 m logs, and the resonance velocity of each log was measured using the HM200, a resonance acoustic tool that is widely used in the New Zealand forest industry for segregating logs on the basis of stiffness [20]. Acoustic velocities of 4 standing trees per subplot were also measured using a ‘time-of-flight’ acoustic velocity tool.

### Economic analysis of the effects of biosolids application

An analysis of the economic effects of applying biosolids to the Rabbit Island plantation was performed, taking account of both the positive effects of biosolids on tree growth, and the negative effects on wood quality, particularly the effects on acoustic velocity. To model growth rate, the *P. radiata* 300 Index Growth Model [21] as implemented in the Radiata Pine Calculator [22] was used. This growth model uses a volume productivity index to account for local site variation, where the index (called the 300 Index) is defined as on the volume mean annual increment at age 30 years for a standard regime (a pruned and early thinned regime with final crop stocking of 300 stems ha^-1^). The model can be used to estimate the 300 Index from a plot measurement (tree heights, DBHs and stocking density) along with the historic stocking, thinning and pruning history of the plot. The model uses an iterative method to estimate the 300 Index consistent with the plot measurement and history. The Radiata Pine Calculator can also be used to estimate the Site Index (defined as the MTH at age 20 years) from a plot measurement using a height/age function. The 300 Index model uses both the volume index (300 Index) and height index (Site Index) to predict growth in a stand for any given management regime. The 300 Index and Site Index were estimated for each plot using the age 18 plot measurement. An analysis of variance was then performed using the SAS Version 9.0 MIXED procedure to test the effect of biosolids loading and stocking treatment on 300 Index and Site Index. To account for pre-existing differences between plots, these analyses used the 300 Index or Site Index estimated from plot measurements made when the trial was established (age 6 years) as a covariate.

The 300 Index growth model was then used to predict stem volume at a harvest age of 30 years for each treatment, using the mean 300 Index and Site Index for each treatment. A common regime was used for all treatments with a stocking at planting of 850 stems ha^-1^, pruning in 3 lifts to 6 m pruned height, and waste thinning to 350 stems ha^-1^ at age 9 years. The Radiata Pine Calculator predicts per hectare harvest volumes for user-defined log grades specified by log length, small end diameter (SED), maximum branch size, and pruning status (pruned or unpruned). However, the Calculator does not currently allow grading by acoustic velocity. The prediction of volumes by grade was therefore performed in two steps. Firstly, the Calculator was used to predict volumes in grades defined on the basis of log size (length and SED), pruning status, and maximum branch size for unpruned logs. The grade specifications used were similar to those in current use (Table 1). Secondly, the volume in each unpruned log grade predicted by the Calculator was apportioned into acoustic velocity classes (<3, 3–3.3, and >3.3 km s^-1^). This was performed assuming that acoustic velocity was normally distributed with mean 3.3 km s^-1^ in the standard treatment (based on the current average of logs harvested from unfertilised stands in the forest), and a coefficient of variation of 6.5% (obtained from the sample logs cut from the stand at age 15 years). For the standard and high biosolids treatments, the mean velocity was adjusted downward to reflect the percentage reductions in velocity observed for each treatment in the trees sampled at age 15 years. Logs predicted to be undersized, heavily swept, or with large branches were graded into a low value pulp grade. Pruned log grades were not subdivided into acoustic velocity grades as they are suited to appearance rather than structural end uses. Prices similar to those currently valid for comparable grades were assigned to each grade. The residual value of each treatment (stumpage) was then calculated based on a fixed harvesting cost of $33 m^-3^. Economic returns expressed as internal rate of return (IRR) and net present value (NPV) were calculated for each treatment using the default Radiata Pine Calculator costs.

**Table 1:** Specifications of log grades used in the study.

Grade	SED a (cm)	Length(m)	Maximum branch diameter (cm)	Acoustic velocity (km s^-1^)	Value ($ m^-3^)
Large pruned	>35	4.9–6.1			133
Small pruned	30–35	3.7–6.1			100
Large unpruned high velocity	>30	4–5.5	15	>3.3	95
Large unpruned medium velocity	>30	4–5.5	15	3.0–3.3	90
Large unpruned low velocity	>30	4–5.5	15	<3.0	75
Small unpruned high velocity	20–30	4–5.5	15	>3.3	85
Small unpruned medium velocity	20–30	4–5.5	15	3.0–3.3	80
Small unpruned low velocity	20–30	4–5.5	15	<3.0	65
Pulp	>10	4–5.5			40

a SED: small end diameter.

## Results and Discussion

**Figure 1:**
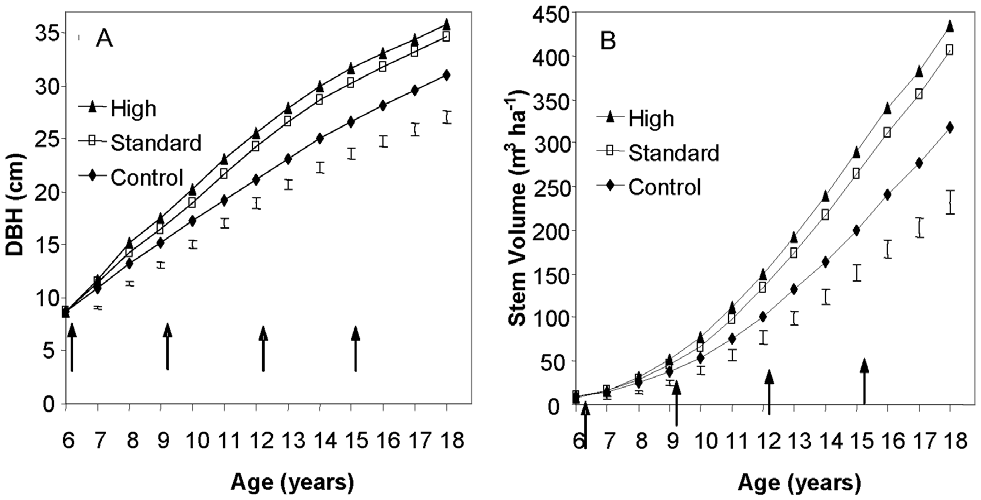
Effect of biosolids application on growth. Measurement of tree (A) diameter at breast height (DBH), and (B) stem volume since the initial biosolids application at age 6 years. The bars represent the least significant difference (LSD) values calculated for each age. Treatment differences greater than the LSD are statistically significant (p  =  0.05). The arrows show when biosolids treatments were applied.

**Figure 2:**
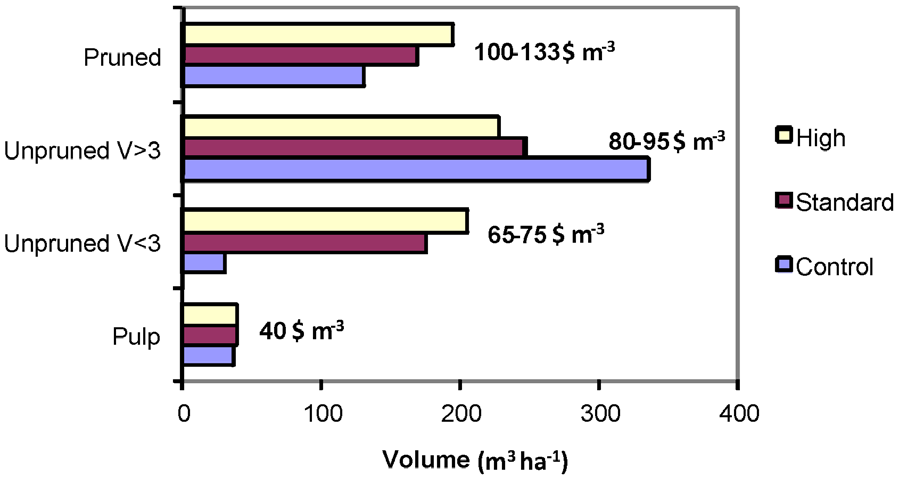
Effect of biosolids application on site productivity. Increase in 300 Index of trees treated with standard and high rates of biosolids versus untreated trees. The bars represent the least significant difference (LSD) values calculated for each age. Differences between the standard and high treatments, and between either of these and the control (zero line), which are greater than the LSD, are statistically significant (p  =  0.05). The arrows show when biosolids treatments were applied.

Annual tree growth measurements show that diameter at breast height (DBH) and stem volume of the pine trees have increased significantly in plots with biosolids since the initial application (Figure 1). In 2009 at age 18, the stem volume of the high treatment (434 m^3^ ha^-1^) was 36% greater than the control (318 m^3^ ha^-1^), and the standard treatment (406 m^3^ ha^-1^) was 27% greater than the control, indicating a substantial gain in productivity. This productivity gain is reflected in the 300 Index, a productivity index based on stem volume mean annual increment. The difference in 300 Index between treated and control plots increased rapidly following the initial biosolids application at age 6 years, but this increase has gradually slowed and the difference appears to have stabilised at about age 14 years (Figure 2). Based on the age 18 year measurement, the 300 Index has been increased from 19.8 m^3^ ha^-1^ yr^-1^ in the control treatment to 23.6 and 25.0 m^3^ ha^-1^ yr^-1^ in the standard and high treatments respectively (Table 2). Site Index, an index of height growth, has only increased slightly in biosolids treatments demonstrating that the principal effect of biosolids has been to increase diameter rather than height growth. Effectively, the biosolids treatments have converted this site from low to medium productivity. The trial mean 300 Indices of the control corresponds to the 20^th^ percentile of a nationwide database of *P. radiata* permanent sample plots maintained by Scion Research, while the standard and high treatments correspond to the 35^th^ and 45^th^ percentiles respectively.

**Table 2:** Productivity indices based on age 18 year measurement, and mean acoustic velocity in standing trees assessed at age 15 years, and resonance velocity assessed in felled trees by log height class at age 15 years, for each biosolids treatment.

	Site Index (m)	300 Index (m^3^ ha^-1^ yr^-1^)	Mean acoustic velocity (km s^-1^)			
			Standing tree	1^st^ log	2^nd^ log	3^rd^ log
Control	30.7 a	19.8 a	3.03 a	2.94 a	2.78 a	2.54 a
Standard	31.6 b	23.6 b	2.79 b	2.70 b	2.59 b	2.33 b
High	31.6 b	25.0 c	2.71 b	2.66 b	2.52 b	2.36 b

Values in a column followed by the same letter do not differ significantly (least significant difference test, p = 0.05).

However, the increased growth has led to some adverse changes in wood quality of the receiving trees including a reduction in wood stiffness. Mean resonance velocity in trees felled at age 15 years was reduced by 8% and 9% in the standard and high treatments respectively compared with the control, with nearly identical reductions observed in the sonic velocities of standing trees (Table 2). The reductions in sonic velocity in treated trees were slightly greater than those observed at age 13 years when reductions of 5% and 7% for standard and high treatments respectively were obtained [10].

The assumed means and standard deviations of resonance velocity of logs at harvest, and the percentage volumes falling into each acoustic class based on the normal distribution are summarised in Table 3. Only 8% of log volume in the control treatment is predicted to fall below the 3 km s^-1^ threshold, but 41–47 % of the log volume in biosolids treated trees is predicted to fall below this threshold.

**Table 3:** Predicted mean acoustic velocity and percentage of logs in acoustic velocity classes for each treatment.

Parameter	Biosolids treatment		
	Control	Standard	High
Mean velocity (km s^-1^)	3.30	3.04	3.01
% Volume velocity > 3.3 km s^-1^	50	10	7
% Volume velocity 3–3.3 km s^-1^	42	49	45
% Volume velocity < 3 km s^-1^	8	41	47

**Figure 3:**
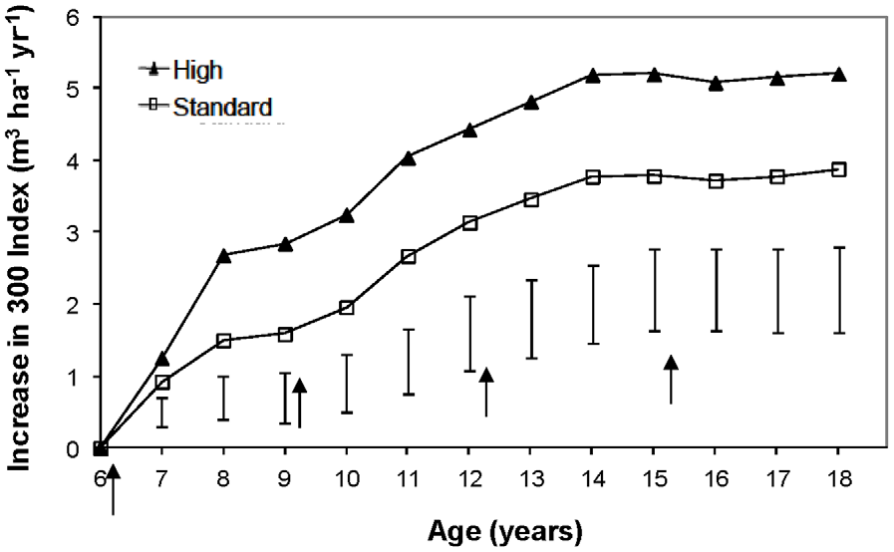
Mean volumes in broad log grade groupings for each biosolids treatment.

The predicted per hectare volumes in each grade at harvest (age 30 years) are shown in Table 4 and further summarised into broad log grade groupings in Figure 3. Predicted values in each grade and the stumpages are shown in Table 5. Overall the high and standard biosolids treatments are predicted to increase the net stumpage value of logs by 24% and 16% respectively at harvesting, providing a large positive impact on the forest owner’s economic return. This analysis takes no account of the expected lower harvesting costs per cubic metre resulting from the larger mean piece size in the biosolids treated trees.

**Table 4:** Predicted harvest volume (m^3^ ha^-1^) by log grade for each biosolids treatment.

Log grade	Biosolids treatment		
	Control	Standard	High
Large pruned	75.5	111.9	138.8
Small pruned	55.4	57.9	55.3
Large unpruned low velocity	9.2	73.9	100.6
Large unpruned medium velocity	47.4	87.1	96.6
Large unpruned high velocity	56.6	17.2	15.1
Small unpruned low velocity	20.3	100.8	105.1
Small unpruned medium velocity	105.3	118.8	100.9
Small unpruned high velocity	125.6	23.5	15.8
Pulp	36.8	39.4	38.7
Total	532.1	630.6	667.0

**Table 5:** Predicted stumpage value ($ ha^-1^) by log grade and across all grades for each biosolids treatment.

Log grade	Biosolids treatment		
	Control	Standard	High
Large pruned	10,045	14,884	18,458
Small pruned	5,542	5,787	5,533
Large unpruned low velocity	687	5,546	7,546
Large unpruned medium velocity	4,268	7,843	8,696
Large unpruned high velocity	5,376	1,635	1,439
Small unpruned low velocity	1,322	6,552	6,830
Small unpruned medium velocity	8,422	9,502	8,072
Small unpruned high velocity	10,677	1,994	1,345
Pulp	1,471	1,577	1,549
Total	47,811	55,321	59,469
Logging cost	–17,561	–20,808	–22,012
Stumpage	30,250	34,513	37,457

The biosolids treatments are predicted to produce much higher volumes of pruned log grades than the control treatment, partly because a greater proportion of pruned logs are expected to exceed the 30 cm minimum SED of the smaller pruned log grade (Table 4). Pruned logs are predicted to be not only of greater total volume, but of greater average value per cubic metre because of the larger proportion of > 35 cm SED logs (Table 6). However, it is predicted that biosolids treatment will cause a greater proportion of unpruned logs to fall into lower acoustic velocity classes (Table 4), and that average per cubic meter value of unpruned logs will be decreased. Despite this, because of the increase in total volume, biosolids treatment is predicted to increase the total value of unpruned logs. Overall, the average value per cubic metre of the combined pruned and unpruned logs is predicted to vary only marginally between the three treatments, with estimates of 90, 88, and 89 $ m^-3^ in the control, standard and high treatment respectively (Table 6).

**Table 6:** Predicted mean value ($ m^-3^) of pruned, unpruned, and all logs, and net mean value of all logs after accounting for harvesting costs in each biosolids treatment.

Log grouping	Biosolids treatment		
	Control	Standard	High
Pruned logs	119.0	121.8	123.6
Unpruned logs	80.3	75.2	75.0
All logs	89.8	87.7	89.2
Net value, all logs	56.8	54.7	56.2

Predicted economic returns in terms of NPV and IRR are shown for each treatment in Table 7. These use default Radiata Pine Calculator cost inputs, and the predicted volumes, log prices and harvesting costs given above. The NPV is calculated for the start of a rotation using a 7% discount rate and takes no account of land value. It can be considered the breakeven value of the land based on the net revenue at harvest, ignoring any change in real value of the land over the course of the rotation, and assuming that the forest owner does not pay for the biosolids treatment. The effect of the treatment is to increase the NPV by about $480 per hectare for the standard rate, and $840 per hectare for the high rate. These values are greater than earlier reported values of $217 and $411 per hectare for the standard and high treatments respectively [17], which were based on the increased growth observed at age 11 years but took no account of any additional increased growth beyond this age. The results reported in the current study are based on age 18 year growth measurements and include the effect of continued increase in growth rate that occurred between ages 11 and 18 years. As the difference in the 300 Index between control and treated trees appears to have stabilised at about age 14 years, the results presented in this paper are expected to be close to those that will ultimately be achieved at harvest, apart from effects of changes in costs or log prices.

**Table 7:** Predicted economic returns over a rotation for each treatment.

Economic result	Biosolids treatment		
	Control	Standard	High
NPV ($ ha^-1^, 7% discount rate) a	1,718	2,202	2,560
IRR (%, excluding land value) b	9.41	9.85	10.15
IRR (%, land value = $5000 ha^-1^) b	5.32	5.71	5.96

a NPV: net present value.

b IRR: internal rate of return.

By multiplying the predicted increase in value per hectare of the standard treatment with the 750-ha biosolids application area, harvest stumpage for the forest is predicted to be increased by $3,200,000, while the estimated NPV at the start of the rotation is improved by $360,000. As the sewerage system and the forest are both owned by the local community, this increased forest value belongs to the community and at least partly defrays the cost of operating the wastewater treatment plant. The operational cost of biosolids management for the scheme is about $450,000 year^-1^ or about 6.5% of total operational costs [23], and over a 30 year rotation these costs would sum to about $14,000,000 while their discounted value at the start of the rotation would be $4,000,000. Therefore, the increase in forest value covers only a fraction of the costs of biosolids management. However, at the time the scheme was designed, forest-based application was seen to be both environmentally and economically the most acceptable solution to the management of biosolids, taking no account of any economic benefit to the forest. The increase in forest value can therefore be seen as a welcome bonus to the community.

It is useful to compare the increase in forest value from applying biosolids with the cost of achieving similar results using conventional N fertiliser. In 2013, the cost of urea fertiliser (46% N) in New Zealand is about of $745 t^-1^ and aerial application costs about $75 t^-1^. This means that the cost of a single application of 300 kg N ha^-1^ using urea is about $535 ha^-1^. When discounted at 7% to the start of the rotation, the cost of multiple applications at ages 6, 9, 12 and 15 years (equivalent to the standard biosolids treatment) is $1080 ha^-1^, more than twice the predicted increase in discounted stumpage value of $484 ha^-1^, and a breakeven return is only achieved at a discount rate of 3.6%. In practice, lower application rates at less frequent intervals might be more economically viable. For example, the discounted cost of a single application of 200 kg N ha^-1^ at age 10 years is $181 ha^-1^, while discounted costs of 2 and 3 applications of this rate between ages 6 and 15 years are about $370 and $550 ha^-1^ respectively. The cost of the latter treatment, which is significantly short of the standard biosolids treatment in terms of N, is slightly greater than the projected increase in discounted log value for the standard treatment, suggesting that the economics of conventional N fertilising on this site are marginal at current fertiliser and log prices.

## Conclusions

Repeated biosolids applications to the *P. radiata* plantation established on this low fertility site low fertility have greatly increased tree growth. At age 18 years, stem volume of the high treatment was 36% greater than the control, and that of the standard treatment was 27% greater than the control. Effectively biosolids have transformed the site from low productivity to medium productivity. Although the increased productivity has been accompanied by some negative influences on wood quality attributes with reduced wood stiffness and density, and larger branches, an economic analysis indicates that the increased stem volume and greater average log diameter in the biosolids treatments outweighs any negative effects on log value due to the reduced stiffness. The high and standard biosolids treatments are predicted to increase the net stumpage value of logs by 24% and 14% respectively at harvesting, providing a large positive impact on the forest owner’s economic return.
